# Point-of-Care Echocardiographic Characteristics of COVID-19 Patients with Pulmonary Embolism

**DOI:** 10.3390/diagnostics12102380

**Published:** 2022-09-30

**Authors:** Slobodan Klasnja, Andrea Manojlovic, Viseslav Popadic, Tatjana Ivankovic, Nebojsa Ninkovic, Nina Rajovic, Maja Popovic, Novica Nikolic, Milica Brajkovic, Aleksandra Radojevic, Ratko Lasica, Sasa Rajsic, Zoran Todorovic, Marija Brankovic, Tijana Radonjic, Lidija Memon, Davor Mrda, Natasa Milic, Marija Zdravkovic

**Affiliations:** 1 University Hospital Medical Center Bežanijska kosa, 11000 Belgrade, Serbia; 2 Institute for Medical Statistics and Informatics, Faculty of Medicine, University of Belgrade, 11000 Belgrade, Serbia; 3 University Clinical Center of Serbia, 11000 Belgrade, Serbia; 4 Faculty of Medicine, University of Belgrade, 11000 Belgrade, Serbia; 5 Department of Anaesthesiology and Intensive Care Medicine, Medical University Innsbruck, 6020 Innsbruck, Austria; 6 Department of Internal Medicine, Division of Nephrology and Hypertension, Mayo Clinic, Rochester, MN 55905, USA

**Keywords:** COVID-19, pulmonary embolism, echocardiography, POCUS

## Abstract

Introduction: Thrombotic complications, such as pulmonary embolism, are common in COVID-19 patients. Point-of-care ultrasound is a highly recommended tool for orientation in critically ill patients with suspected or confirmed complications. Methods: An observational study was conducted on 32 consecutive patients with confirmed pulmonary embolism and COVID-19 infection treated in the Intensive Care Unit of the University Hospital Medical Center “Bežanijska kosa”, Belgrade, Serbia, between April 2021 and March 2022. Predictors of the need for oxygen support were determined, while point-of-care echocardiographic parameters and various anamnestic, laboratory, and clinically significant parameters were correlated with the Pulmonary Embolism Severity Index (PESI) score. Results: More than two-thirds of patients in our study had PE symptoms present at hospital admission (68.8%). The majority of patients had segmental pulmonary embolism (48.4%), with high to very high PESI score values in 31.3% of patients. Pneumonia was present in 68.8% of the study population. The PESI score was negatively correlated with diastolic blood pressure and SaO2 at the time of PE diagnosis, LV ejection fraction, and PVAT. A positive correlation was found between the PESI score, maximum CRP, and D-dimer at the time of PTE diagnosis. A larger right ventricular diameter was associated with a greater need for oxygen support. Conclusion: Point-of-care echocardiography is a valuable tool for the risk assessment of COVID-19 patients with pulmonary embolism. Right ventricular size stood out as a significant marker of disease severity.

## 1. Introduction

For almost three years, the medical society has been dealing with the implications and consequences of the novel, highly transmissible, and virulent disease COVID-19, with patients ranging from critically ill to seemingly asymptomatic. The most severely ill patients with respiratory failure often seemed to deteriorate more rapidly than expected, a discrepancy that was presumed to be due to thrombotic complications [1]. The finding that initially pointed in that direction was serum D-dimer, which was positively correlated with poor prognosis in critically ill patients early on in the pandemic [2,3,4]. D-dimer is a degradation product of fibrinolysis, a distinctly sensitive marker but not specific to thrombosis, as there are various conditions that can influence it [5]. Nevertheless, a significant increase in serum D-dimer levels likely reflects intravascular thrombosis in patients with COVID-19. COVID-19 is characterized by other coagulation abnormalities, such as a dramatic increase in thrombin production and elevated concentrations of both von Willebrand factor and factor V (6). Besides hypercoagulability, endothelial injury was identified as an adjacent pathophysiological factor in thrombotic complications. Taking into account that the in vivo biosynthesis of von Willebrand factor is restricted to endothelial cells and megakaryocytes, high plasma von Willebrand factor concentrations in patients with COVID-19 suggest significant endothelial cell injury [6]. Furthermore, certain studies reported that, of the critically ill patients with COVID-19 found to have pulmonary thrombosis via screening computerized tomography pulmonary angiogram (CTPA), only a small percentage had deep vein thrombosis as a cause of thromboembolism [7]. These findings, including the fact that we commonly see segmental and subsegmental pulmonary thrombosis, as described on CTPA, have sparked a debate: Can pulmonary embolism (PE) in COVID-19 be explained by the same mechanisms as PE in non-COVID-19 patients, or do we mainly consider intravascular thrombosis of the pulmonary artery and its branches? The incidence of PE in COVID-19 patients varies depending on the severity of the disease and, in multiple studies and meta-analyses, is reported to be between 11% and 21%, with the incidence in Intensive Care Unit (ICU) patients being higher than that in non-ICU patients [8,9]. An important observation is that the majority of patients have been receiving at least prophylactic doses of anticoagulation therapy prior to PE diagnosis, which means that proper anticoagulation does not exclude thrombotic complications as a reason for clinical deterioration, so further diagnostic methods should follow clinical suspicion. Considering that SARS-CoV-2 is highly contagious, diagnostic procedures should be brief but still effective in monitoring cardiac and pulmonary function and assessing possible complications. Point-of-care ultrasonography (POCUS) is defined as ultrasonography brought to the patient and performed by the provider in real time [10], and it is a highly recommended tool for orientation [11,12]. The following paper analyzes the POCUS characteristics of COVID-19 patients with verified pulmonary embolism and their correlation with disease severity.

## 2. Materials and Methods

### 2.1. Study Design

**Figure 1:**
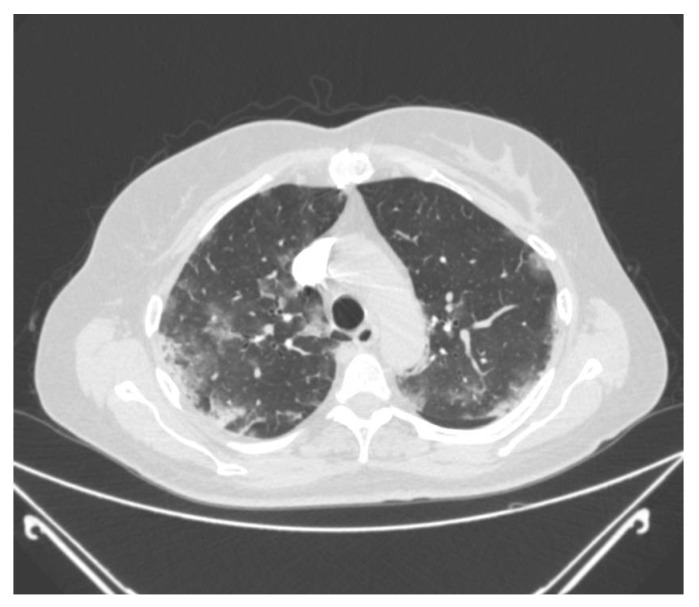
Ground-glass opacities on chest CT in a patient with COVID-19-related pneumonia.

The study was conducted as an observational study on all (32) consecutive patients with confirmed PE and COVID-19 infection treated in the Intensive Care Unit of the University Hospital Medical Center “Bežanijska kosa”, Belgrade, Serbia, between April 2021 and March 2022. COVID-19 infection was confirmed by reverse transcriptase polymerase chain reaction (RT-PCR) from a nasopharyngeal swab sample. PE was diagnosed using the protocol for multi-slice computed tomography (MSCT) pulmonary angiography and was characterized as subsegmental, segmental, lobar, and massive. COVID-19 pneumonia was confirmed radiographically by chest computed tomography (CT). Chest CT was performed on all participants to evaluate the stage and extensiveness of interstitial pneumonia. Typical COVID-19 pneumonia changes were evaluated, including bilateral lung involvement with ground-glass opacities, consolidations, interlobular septal thickening, and crazy paving patterns, as well as pleural effusion and lymphadenopathy (Figure 1). According to the severity of the observed changes, every lobe was given 0–5 points (for each of the upper, middle, and lower right lung lobes and upper and lower left lung lobes), resulting in a maximum possible score of 25 points. In addition to the scoring, the disease was classified into 4 stages (early, progressive, peak, and resolution). The main clinical criteria for ICU admission were pneumonia progression on radiographic or chest CT examination, peripheral oxygen saturation (Sp02) below 93% despite maximal conventional supportive oxygen therapy (through a nasal cannula, conventional oxygen, or non-rebreathing mask), and further impairment according to arterial blood gas test and laboratory test results, mainly an increase in inflammatory parameters. Color Doppler imaging of the lower extremities was performed in all participants upon PE diagnosis.

### 2.2. Demographic Data, Past Medical History, and Laboratory Parameters

Demographic data (age, gender, and BMI), past medical history (hypertension, diabetes mellitus, hyperlipidemia, coronary heart disease, chronic obstructive pulmonary disease (COPD), previous history of deep vein thrombosis and pulmonary embolism, and prior immobilization), history of smoking, and vaccination status were collected. Laboratory parameters upon admission to the hospital were collected, including the values of urea, glycemia, bilirubin, alanine (ALT) and aspartate transaminase (AST), lactate dehydrogenase (LDH), creatine kinase (CK), gamma-glutamyl transferase (GGT), potassium, sodium, magnesium, chloride, amylase, creatinine, uric acid, serum albumins, serum proteins, C-reactive protein (CRP), procalcitonin, triglycerides, total cholesterol, complete blood count, high-sensitivity troponin T (hsTnT), N-terminal pro-brain natriuretic peptide (NT-proBNP), D-dimer, interleukin-6, prothrombin time, activated partial thromboplastin time, and fibrinogen. The maximum values of D-dimer, IL-6, CRP, and procalcitonin were also followed during hospitalization. The reference values of the evaluated laboratory parameters are presented in Supplemental Table S1.

### 2.3. Clinically Significant Parameters and Outcomes

Clinical parameters upon admission and at the time of PE diagnosis were collected, including the number of days from symptom onset until hospital admission and until the diagnosis of PE, peripheral oxygen saturation, heart rate, blood pressure, and body temperature. The Pulmonary Embolism Severity Index (PESI) score to determine the risk of mortality and the severity of complications was calculated for every patient, as recommended in the 2019 ESC Guidelines for the Diagnosis and Management of Acute PE [13]. The score criteria and risk classes are provided in Supplemental Table S2. The need for oxygen support and mortality were considered the primary outcomes.

### 2.4. Echocardiography

**Figure 2:**
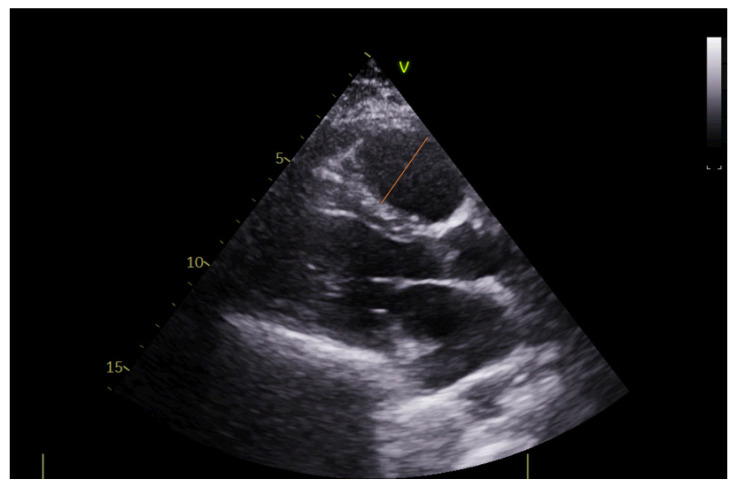
Right ventricular diameter from the parasternal long-axis (PLAX) view.

**Figure 3:**
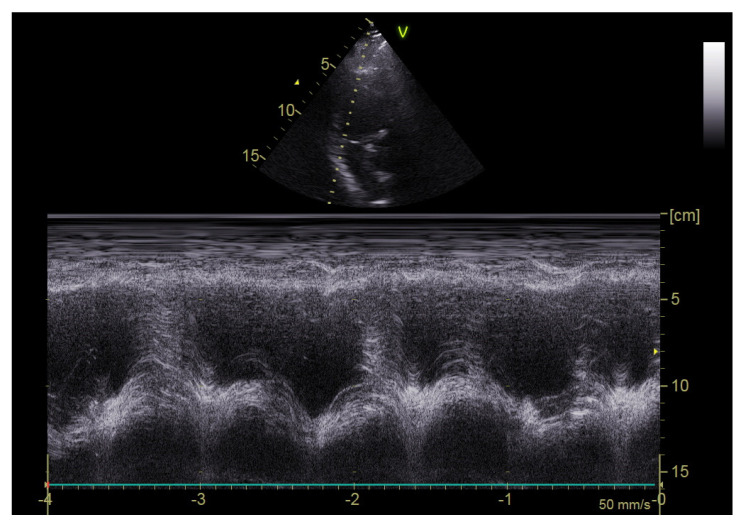
M mode of TAPSE of the free right ventricular wall as seen from the right-ventricle-focused apical view.

**Figure 4:**
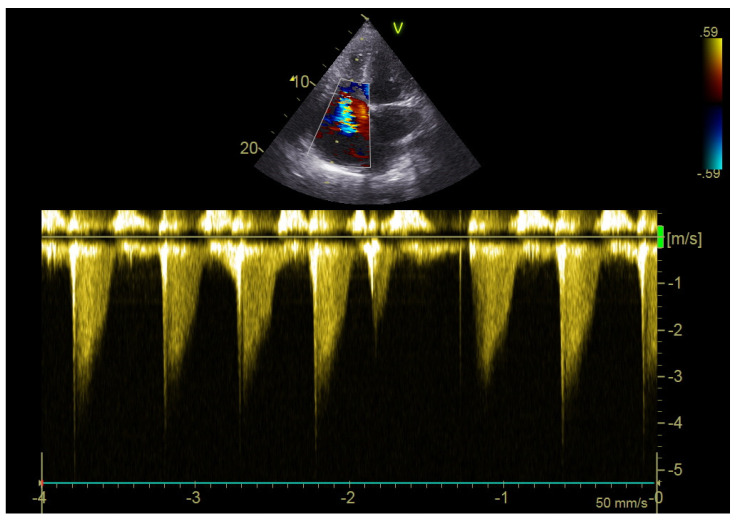
Continuous-Wave (CW) Doppler of tricuspid regurgitation from the 4-chamber view.

The echocardiographic study was performed on a portable echocardiography machine (Vivid Iq, GE Healthcare, Chicago, IL, USA) by two cardiologists with more than 20 years of experience in echocardiography in all participants upon their admission to the ICU and after a confirmed diagnosis of PE by CT pulmonary angiography. The probe 3Sc-RS (1.3–4.0 Mhz) with variable depth during the examination (mainly 10 cm depth) was used. Various echocardiographic parameters were evaluated, including left ventricular ejection fraction, right ventricular diameter, right/left ventricular diameter ratio, right atrial area, Tricuspid Annular Plane Systolic Excursion (TAPSE), lateral tricuspid annulus peak systolic velocity (S’ RV), right ventricular end-systolic pressure (RVESP), inferior vena cava diameter (VCI), pulmonary velocity acceleration time (PVAT), and the presence of McConnell’s sign (right ventricular free wall akinesia with sparing of the apex) (Figure 2, Figure 3 and Figure 4). The normal values of the evaluated echocardiographic parameters are presented in Supplemental Table S3.

All patients were managed with supportive care and specific pharmacological protocols created by the hospital’s COVID-19 management guidelines committee in accordance with the Ministry of Health, the Republic of Serbia. PE was treated according to the 2019 guidelines for the diagnosis and management of acute pulmonary embolism recommended by the European Society of Cardiology [13]. The duration of ICU stay, the need for oxygen support, including non-invasive and mechanical ventilation, and in-hospital mortality were followed as outcomes in all participants.

### 2.5. Statistical Analysis

Numerical data are presented as means with standard deviations or medians with percentiles. Categorical variables are summarized by absolute numbers with percentages. Differences in the need for oxygen support and patients’ clinical, laboratory, and point-of-care echocardiographic parameters were analyzed using the Chi-Square test, Students’ test for independent samples, or the Mann–Whitney U test. Correlations between the PESI score and patients’ clinical, laboratory, and echocardiographic parameters were assessed by the Spearman correlation coefficient. Univariate and multivariate logistic regression models were used to assess the predictors of the need for oxygen support (as a dependent variable). In all analyses, the significance level was set at 0.05. Statistical analysis was performed using IBM SPSS statistical software (SPSS for Windows, release 25.0, SPSS, Chicago, IL, USA).

## 3. Results

A total of 32 patients with PE and confirmed COVID-19 infection were included in the study. More than half of the patients were male (53.1%), with a mean age of 64.13 ± 17.3 years. Hypertension was the most frequent comorbidity (65.6%), diabetes mellitus type 2 was present in 12.5%, and deep vein thrombosis was present in 12.5% of patients. Additional demographic data and the comorbidities of the study population are presented in Table 1.

The clinical parameters of the study population are shown in Table 2. More than two-thirds of patients had PE symptoms present at hospital admission (68.8%). The majority of patients had segmental PE (48.4%), with high to very high PESI score values in 31.3% of patients. Pneumonia was present in 68.8% of the study population, mostly in the peak stage, as estimated by chest CT (34.6%). The median CT severity score was 8, and 62.5% of patients in the study population were in need of oxygen, of which 12.5% were intubated. Four patients had a fatal outcome (12.5%) (Table 2).

The laboratory parameters of the study population are presented in Table 3.

At the time of PE diagnosis, the mean heart rate of the study population was 88 ± 23 beats per minute, with a mean systolic and diastolic blood pressure of 125 ± 22 mmHg and 78 ± 13 mmHg, respectively. The median value of D-dimer was 6811 (25th–75th percentile: 3428–14,128), while the mean oxygen saturation of the study population was 93 ± 7 percent (Table 4).

In Table 5, the point-of-care echocardiographic parameters of the study population are presented.

Older patients and patients with an intermediate/high or very high PESI score, higher values of CRP, a higher CT severity score, higher values of D-dimer, lower SaO2 at the time of PE diagnosis, and a larger right ventricular diameter were more often in need of oxygen support. Patients in whom PE symptoms were present at hospital admission needed oxygen support less often (Table 6).

The correlation between the PESI score and the laboratory parameters, clinical parameters, main parameters at the time of PE diagnosis, and point-of-care echocardiographic parameters of the study population are presented in Table 6. The PESI score was negatively correlated with diastolic blood pressure (rho = 0.357; *p* = 0.049), SaO2 at the time of PE diagnosis (rho = 0.366; *p* = 0.043), LV ejection fraction (rho = 0.387; *p* = 0.028), and PVAT (rho = 0.535; *p* = 0.049). A positive correlation was found between the PESI score and CRP max (rho = 0.445; *p* = 0.011), as well as with values of D-dimer at the time of PE diagnosis (rho = 0.388; *p* = 0.031) (Table 7).

In Table 8, univariate and multivariate logistic regression analyses, with the need for oxygen support as a dependent variable, are presented. Older patients (*p* = 0.013), higher PESI score (*p* = 0.014), higher CT score (*p* = 0.005), higher values of D-dimer (*p* = 0.041), and right ventricular diameter (*p* = 0.030) were significant predictors of the need for oxygen support in univariate logistic regression analysis. Higher PESI (*p* = 0.042) and CT scores (*p* = 0.035) were significant independent predictors of polyp recurrence in multivariate logistic regression analysis (Table 8).

Patients who were on anticoagulant therapy more often had higher PESI scores (81.8%) in comparison to patients who were not on anticoagulant therapy before diagnosis (33.3%) (*p* = 0.009). In addition, borderline significance was found in PVAT values, where patients who were on anticoagulant therapy more often had lower values of PVAT (*p* = 0.053).

## 4. Discussion

In the present study of 32 consecutive patients with confirmed pulmonary embolism and COVID-19 infection, patients with a larger right ventricular diameter required oxygen support more often, which was correlated with the PESI score as an indicator of PE severity. It is notable that COVID-19 patients presenting primarily with PE usually belonged to the low-risk group according to the calculated PESI score and rarely progressed to right ventricular dysfunction and hemodynamic instability. Already-hospitalized patients with severe COVID-19 pneumonia who developed PE during their hospitalization had worse outcomes, probably due to several pathophysiological mechanisms involved in the pathogenesis of PE.

In the first months of the pandemic, it became clear that fast decision-making would determine the course of the disease, so diagnostic methods and guidelines were rapidly put in place to enhance efficiency. Diagnostics had to be precise but also involve a minimum duration of contact between the physician and patient to reduce the possibility of disease transmission. Point-of-care ultrasound is recommended for evaluating critically ill patients with respiratory failure, multisystem organ failure, or shock. Its advantages compared with traditional imaging modalities are rapid assessment of a patient’s change in status, easy bedside access with no need to transport already-critical patients on multi-organ support machines, and decreased potential for the transmission of SARS-CoV-2 to staff and others during transport [14]. In our scans, the ultrasound was performed in the usual echocardiographic views—short and long parasternal axes, apical four-, two-, and three-chamber views, and the subcostal view. The parameters that were obtained (listed in Supplementary Table S3) varied depending on the clinical aspect and the severity of the patient’s condition. As recommended, the goal was to assess the size and function of both the left and right ventricles, the presence of pericardial effusion, and indirectly measured right-chamber and pulmonary pressures to determine the cause of the deterioration of the patient’s condition [15]. Taking into account that a significant percentage of critically ill patients have comorbidities, such as COPD or congestive heart failure, it is of undeniable importance to determine the cause of respiratory failure to apply a treatment plan [16]. Congestive heart failure exacerbation, COPD exacerbation, COVID-19 acute respiratory distress syndrome (ARDS), and PE are common considerations in this patient population [17]. Keeping in mind that more than half of the patients in our cohort had low-risk PE, without affecting the large pulmonary artery branches, the values of the functional parameters of the right ventricle were mostly in the physiological range. Nevertheless, a larger right ventricular diameter, as an indirect indicator of elevated pulmonary vascular pressure, was positively correlated with the need for oxygen support. This information might be crucial, as it isolates patients with a higher risk of deterioration, who can then be more closely monitored and in whom complications might be prevented. Lower (pathological) levels of PVAT were found in patients with PE who were already on anticoagulation therapy. These patients were mostly those who were already hospitalized with pneumonia and severe COVID-19 with higher PESI scores. Bearing in mind that these patients already had increased pulmonary pressures due to interstitial lung consolidations and, not rarely, were on mechanical ventilatory support, PVAT might point to an acute rise in pulmonary artery pressure as a sign of acute PE. Another study considering echocardiographic abnormalities and the connection to worse outcomes in COVID-19 patients is described in a paper by Vincenza Polito et al. [18]. They concluded that RV systolic dysfunction, high pulmonary pressures, and poor RV–arterial coupling independently predict the risk of mortality and PE in hospitalized patients with COVID-19, both in the ICU and the ward. While the advantage of this study in comparison to ours was a larger cohort of patients (227 compared to 32), our study is novel in including only patients hospitalized with COVID-19 and confirmed PE by MSCT pulmonary angiography, as opposed to all hospitalized COVID-19 patients. Nevertheless, the mentioned study also showed significance in the early echocardiographic evaluation of patients and risk stratification.

Aside from POCUS, as an important method to quickly estimate a patient’s condition, the close monitoring of possible PE causes is of great importance, with deep vein thrombosis as one of the most significant. Deep vein thrombosis was identified as a source of emboli based on clinical findings or color Doppler ultrasound in only 12.5% of all of our patients, which is in accordance with several other reports, such as Mirsadraee et al. [6], where only 23% of patients with a CTPA diagnosis of PE also had radiological evidence of peripheral deep vein thrombosis. Keeping this in mind, thrombotic complications of COVID-19 are more likely explained by a cluster of pathophysiological mechanisms and not only as a consequence of thromboembolism. Hemostatic changes occur in SARS-CoV-2 infection, which might be a specific effect of SARS-CoV-2 or a consequence of a cytokine storm, as observed in other viral diseases. Systemic inflammation can then be followed by disseminated intravascular coagulation, which may predispose COVID-19 patients to micro- and macrovascular pulmonary thrombosis. The local endothelial injury of pulmonary blood vessels by the virus itself or inflammatory cytokines forms predilection sites for in situ thrombosis [19]. The potential interaction of novel antiviral and biological therapies (monoclonal antibodies), as well as other pharmacological agents being used in the treatment of COVID-19 patients, could disrupt the effect of anticoagulation therapy, which would explain the high prevalence of thrombotic complications in patients already on anticoagulation therapy [20]. Lastly, patients with the most severe form of the disease on mechanical ventilatory support are bedridden and immobilized. A total of 10 (32.2%) patients from our cohort were diagnosed with PE during hospitalization, and all of them were already on anticoagulation therapy, although these were also the patients with the most severe forms of the disease. This points out the potential significance of multiple pathophysiological mechanisms being involved in thromboembolic complications and strongly affecting the coagulation status, which usually leads to a more severe hemodynamic deterioration and poor clinical outcomes. It is also important to note that pneumonia was present in two-thirds of the patients, which means that 32% of patients with CTPA-verified PE did not have any signs of viral pneumonia on CT. This implies that patients can have severe, life-threatening complications of the disease without respiratory tract involvement, especially in a younger population. This is why a quick and proper diagnostic algorithm can save time and prevent possible severe complications.

The main limitation of this study is the relatively small cohort of patients recruited from a single center. Echocardiography was focused solely on patients with confirmed or suspected complications of the disease, such as PE, in order to minimize unnecessary contact and prevent the disease from spreading. Disregarding the small sample size, this is the first study concerning only COVID-19 patients hospitalized in the ICU with verified PE. Future studies are required to determine whether similar results can be obtained in different clinical settings with a larger sample size. The lack of complete echocardiographic studies for all observed PE patients prevents the generalization of the obtained correlations with the major complications of the disease and poor clinical outcomes.

## 5. Conclusions

COVID-19 can cause a multisystemic disease with numerous complications, PE being one of the most common. In COVID-19 patients with rapid worsening, timely assessment and diagnosis are a priority to lower mortality. POCUS is a quick, safe, and valuable tool for differential diagnosis in the deterioration of critically ill patients and a potential screening method for identifying COVID-19 patients with a higher risk of complications. In terms of point-of-care echocardiography among patients with PE and COVID-19, right ventricular size stood out as a significant marker of disease severity, while shortened PVAT might be indicative of a PE diagnosis as a reason for the acute worsening of a critically ill patient.

## Figures and Tables

**Table 1:** Demographic data and comorbidities of the study population.

Variable	*n* (%)
Gender	
Male	17 (53.1)
Female	15 (46.9)
Age, mean ± sd	64.13 ± 17.3
Smoking, yes	4 (12.5)
Comorbidity	
Cardiovascular diseases	3 (9.4)
Hypertension	21 (65.6)
Heart failure	3 (9.4)
Diabetes mellitus type 2	4 (12.5)
Previous deep vein thrombosis	4 (12.5)
Previous pulmonary embolism	0 (0.0)
Previous immobilization longer than 3 days, yes	3 (9.4)
Current symptoms of deep vein thrombosis, yes	3 (9.4)
Malignancy, yes	5 (15.6)
Vaccination status	
Vaccinated	12 (37.5)
Non-vaccinated	20 (62.5)

**Table 2:** Clinical parameters of the study population.

Variable	*n* (%)
From beginning of symptoms to hospital admission (days), median (25th–75th percentile)	8 (4.5–11.0)
Pulmonary embolism symptoms present at hospital admission, yes	22 (68.8)
Time from hospital admission to pulmonary embolism symptoms (days), median (25th–75th percentile)	8 (1.0–14.2)
Classification based on CT pulmonary angiography findings	
Subsegmental PE	3 (9.7)
Segmental PE	15 (48.4)
Lobar PE	10 (32.3)
Massive PE	3 (9.7)
Pulmonary Embolism Severity Index (PESI) score	
Very low (≤65)	5 (15.6)
Low (66–85)	11 (34.4)
Intermediate (86–105)	6 (18.8)
High (106–125)	6 (18.8)
Very high (≥125)	4 (12.5)
Pneumonia, yes	22 (68.8)
Pneumonia stages verified by chest CT	
Initial stage	5 (19.2)
Progressive stage	7 (26.9)
Peak stage	9 (34.6)
Resolution	5 (19.2)
CT severity score, median (25th–75th percentile)	8 (0.0–16.75)
ICU stay (total days), median (25th–75th percentile)	10 (7.25–14.75)
Anticoagulant therapy, yes	11 (34.4)
Need for oxygen support, yes	20 (62.5)
Non-invasive	16 (50.0)
Mechanical ventilation	4 (12.5)
Fatal outcome, yes	4 (12.5)

**Table 3:** Laboratory parameters of the study population.

Variable	
Leukocytes, median (25th–75th percentile)	6.02 (4.81–9.20)
Neutrophils, median (25th–75th percentile)	4.76 (2.60–6.32)
Lymphocytes, median (25th–75th percentile)	1.03 (0.87–1.48)
Hemoglobin, mean ± sd	129 ± 21
Thrombocytes, median (25th–75th percentile)	196 (146–284)
Urea, median (25th–75th percentile)	5.9 (5.1–8.4)
Creatinine, median (25th–75th percentile)	88 (75–102)
AST, median (25th–75th percentile)	26 (19–38)
ALT, median (25th–75th percentile)	30 (20–46)
D-dimer, median (25th–75th percentile)	5072 (2709–11,527)
D-dimer max, median (25th–75th percentile)	8110 (4241–18,355)
INR, median (25th–75th percentile)	1.05 (1.0–1.10)
aPTT, mean ± sd	23.7± 4.1
NT-ProBNP, median (25th–75th percentile)	1807 (278.4–5549.0)
CRP, median (25th–75th percentile)	26.85 (6.4–42.7)
CRP max, median (25th–75th percentile)	35.85 (21.5–125.8)
IL-6 max, median (25th–75th percentile)	28.9 (9.11–49.6)

**Table 4:** Main parameters at the time of pulmonary embolism diagnosis.

Variable	
Heart rate (/min), mean ± sd	88 ± 23
Systolic blood pressure (mm/Hg), mean ± sd	125 ± 22
Diastolic blood pressure (mm/Hg), mean ± sd	78 ± 13
D-dimer, median (25th–75th percentile)	6811 (3428–14,128)
SaO2 (%), mean ± sd	93 ± 7

**Table 5:** Echocardiographic parameters of the study population.

Variable	
LV ejection fraction (%), mean ± sd	57 ± 10
Right ventricular diameter (mm), mean ± sd	29 ± 6
Right/left ventricular diameter ratio, mean ± sd	0.64 ± 0.11
Right atrial area (cm^2^), mean ± sd	17.3 ± 5.3
TAPSE (mm), mean ± sd	23 ± 4
S’RV (cm/s), mean ± sd	16 ± 3
PVAT (msec), mean ± sd	120 ± 25
RV ESP (mmHg), mean ± sd	36 ± 11
VCI (mm), mean ± sd	15 ± 2
Signs of deep vein thrombosis on color Doppler, yes, *n* (%)	9 (28.1)

**Table 6:** Need for oxygen support according to characteristics of patients, clinical parameters, laboratory parameters, vital parameters at the time of pulmonary embolism diagnosis, and point-of-care echocardiographic parameters of the study population.

Variable	Need for Oxygen Support		*p*
	No (*n* = 12)	Yes (*n* = 20)	
Age, mean ± sd	53.1 ± 21.5	70.7 ± 9.8	**0.003**
Pulmonary embolism symptoms present at hospital admission, yes	12 (100.0)	10 (50.0)	**0.003**
PESI score			
Very low/low	10 (83.3)	6 (30.0)	**0.003**
Intermediate–high/very high	2 (16.7)	14 (70.0)	
CRP max, median (25th–75th percentile)	26.9 (6.2–46.0)	47.0 (28.4–174.7)	**0.045**
CT severity score, median (25th–75th percentile)	0 (0–5)	14 (8–18)	**0.001**
Heart rate (/min), mean ± sd	82.4 ± 20.1	91.6 ± 24.0	0.280
Systolic blood pressure (mm/Hg), mean ± sd	127.1 ± 24.3	122.9 ± 20.6	0.610
Diastolic blood pressure (mm/Hg), mean ± sd	81.7 ± 9.6	76.3 ± 15.1	0.283
D-dimer, median (25th–75th percentile)	3766 (3105–6700)	9590 (5610–20,750)	**0.023**
SaO2 (%), mean ± sd **Point-of-care echocardiographic parameters**	97.3 ± 1.0	90.6 ± 7.6	**0.005**
LV ejection fraction (%), mean ± sd	55.8 ± 14.6	57.8 ± 5.4	0.579
Right ventricular diameter (mm), mean ± sd	26.2 ± 3.5	31.3 ± 5.8	**0.012**
Right/left ventricular diameter ratio, mean ± sd	0.60 ± 0.11	0.66 ± 0.11	0.461
Right atrial area (cm^2^), mean ± sd	17.5 ± 6.7	17.2 ± 4.2	0.922
TAPSE (mm), mean ± sd	21.3 ± 5.1	23.7 ± 2.2	0.112
S’RV (cm/s), mean ± sd	13.2 ± 1.7	17.4 ± 3.4	0.061
PVAT (msec), mean ± sd	131.5 ± 14.0	114.7 ± 27.7	0.278
RV ESP (mmHg), mean ± sd	35.5 ± 13.7	35.7 ± 10.7	0.964

**Table 7:** Correlation of PESI score and characteristics of patients, clinical parameters, laboratory parameters, vital parameters at the time of pulmonary embolism diagnosis, and echocardiographic parameters of the study population.

Variable	PESI Score	
	Rho	*p*
CRP max	0.445	**0.011**
CT severity score	0.292	0.105
Heart rate	0.254	0.168
Systolic blood pressure	−0.328	0.071
Diastolic blood pressure	−0.357	**0.049**
D-dimer at PE diagnosis	0.388	**0.031**
SaO2 at PE diagnosis	−0.366	**0.043**
LV ejection fraction	−0.387	**0.028**
Right ventricular diameter	0.315	0.084
Right/left ventricular diameter ratio	0.014	0.960
Right atrial area	0.192	0.493
TAPSE	−0.161	0.441
S’RV	0.429	0.249
PVAT	−0.535	**0.049**
RV ESP	0.257	0.248
VCI	0.188	0.722

**Table 8:** Univariate and multivariate logistic regression analyses with the need for oxygen support as a dependent variable.

Variable	Univariate			Multivariate		
	*p*	OR	95% CI	*p*	OR	95% CI
Age	0.013	1.075	1.015–1.138			
PESI score	0.014	4.452	1.361–14.560	0.042	4.614	1.057–20.132
CT score	0.005	1.275	1.075–1.513	0.035	1.319	1.020–1.706
D-dimer	0.041	1.000	1.000–1.000			
RV diameter	0.030	1.272	1.023–1.582			

## Data Availability

The data that support the findings of this study are available from the corresponding author (VP) upon reasonable request.

## Supplementary Materials

The following supporting information can be downloaded at: https://www.mdpi.com/article/10.3390/diagnostics12102380/s1. Supplemental Table S1. Reference values of evaluated laboratory parameters; Supplemental Table S2. Pulmonary Embolism Severity Index (PESI) score criteria and risk classes; Supplemental Table S3. Reference values of evaluated echocardiographic parameters.
