# Supplementary material for: Point-of-Care Echocardiographic Characteristics of COVID-19 Patients with Pulmonary Embolism
Source: Diagnostics (Basel). 2022 Sep 30;12(10):2380. doi: 10.3390/diagnostics12102380 (PMC9600333; doi:10.3390/diagnostics12102380)
Supplement: Supplementary file 1 [file diagnostics-12-02380-s001.zip › Supplemental Table S1.pdf]

Supplemental Table S1. Reference values of evaluated laboratory parameters

Parameter	Reference value
Urea	2.8-8.1 mmol/L
Glucose	4.1-5.9 mmol/L
Total bilirubine	up to 21 umol/L
Alanine transaminase (ALT)	up to 41 U/L in men, up to 33 U/L in women
Aspartate transaminase (AST)	up to 40 U/L in men, up to 32 U/L in women
Lactate dehydrogenase (LDH)	270.0-450.0 U/L in men, 270.0-428.0 U/L in women
Creatine kinase (CK)	up to 190 U/L in men, up to 170 U/L in women
Gamma-glutamyltransferase (GGT)	10.0-71.0 U/L in men, 6.0-42.0 U/L in women
Potassium	3.5-5.1 mmol/L
Sodium	136.0-145.0 mmol/L
Chloride	98.0-107.0 mmol/L
Amylase	28.0-100.0 mmol/L
Creatinine	62.0-106.0 umol/L in men, 44.0-80.0 umol/L in women
Uric acid	202.3-416.5 umol/L in men, 142.8-339.2 umol/L in women
Serum albumins	35.0-52.0 g/L
Serum proteins	64.0-83.0 g/L
C-reactive protein	up to 5 mg/L
Triglycerides	up to 1.7 mmol/L
Total cholesterol	up to 5.2 mmol/L
INR	0.9-1.1
PT	82.0-121.0 %
APTT	23.0-31.9 s
Fibrinogen	2.1-4.0 g/L
IL6	0.0-7.0 pg/mL
PCT	0.00-0.05 ng/mL
High sensitive troponin T (hsTnT)	up to 14 ng/L

N-Terminal pro-Brain natriurethic peptide (NT-proBNP)	up to 125 pg/mL
Fibrinogen	2.1-4.0 g/L
**Complete blood count**	
Leukocytes	$4.0\text{-}10.0x10^9/L$
Erythrocytes	$4.5\text{-}6.5/10^{12}/L$ in men, $3.8\text{-}5.8/10^{12}/L$ in women
Hemoglobin	130-170g/L in men, 115-160g/L in women
Hematocrit	0.40-0.54L/L in men, 0.37-0.47L/L in women
Thrombocytes	$150.0\text{-}450.0x10^9/L$
Neutrophils	$2.0\text{-}7.5x10^9/L$
Lymphocytes	$1.0\text{-}4.0x10^9/L$
Monocytes	$0.2\text{-}1.0x10^9/L$
Eosinophils	up to $0.5x10^9/L$
Basophils	up to $0.2x10^9/L$
