# Supplementary material for: Point-of-Care Echocardiographic Characteristics of COVID-19 Patients with Pulmonary Embolism
Source: Diagnostics (Basel). 2022 Sep 30;12(10):2380. doi: 10.3390/diagnostics12102380 (PMC9600333; doi:10.3390/diagnostics12102380)
Supplement: Supplementary file 1 [file diagnostics-12-02380-s001.zip › Supplemental Table S2.pdf]

Supplemental Table S2. Pulmonary Embolism Severity Index (PESI) score criteria and risk classes

Age		
Sex	**Female** 0	**Male** +10
History of cancer	**No** 0	**Yes** +30
History of heart failure	**No** 0	**Yes** +10
History of chronic lung disease	**No** 0	**Yes** +10
Heart rate ≥110	**No** 0	**Yes** +20
Systolic BP <100 mmHg	**No** 0	**Yes** +30
Respiratory rate ≥30	**No** 0	**Yes** +20
Temperature <36°C	**No** 0	**Yes** +20
Altered mental status (disorientation, lethargy, stupor, or coma)	**No** 0	**Yes** +60
O2 saturation <90%	**No** 0	**Yes** +20

**PESI score**	**Class**	**Risk 30 day mortality**
0-65	I	0.0-1.6%
66-85	II	1.7-3.5%
86-105	III	3.2-7.1%
106-125	IV	4.0-11.4%
≥125	V	10-24.5%
