# Supplementary material for: Point-of-Care Echocardiographic Characteristics of COVID-19 Patients with Pulmonary Embolism
Source: Diagnostics (Basel). 2022 Sep 30;12(10):2380. doi: 10.3390/diagnostics12102380 (PMC9600333; doi:10.3390/diagnostics12102380)
Supplement: Supplementary file 1 [file diagnostics-12-02380-s001.zip › Supplemental Table S3.pdf]

Supplemental Table S3. Reference values of evaluated echocardiographic parameters

**Parameter**	**Normal value**
LV ejection fraction	**> 54% F**<br>**> 52% M**
RV size on PLAX	**< 31mm**
RV/LV diametar ratio	**<1**
RA area	**< 18cm2**
TAPSE	**< 17mm**
S'RV	**> 9cm**/s
PVAT (pulmonary acceleration time)	**> 130msec**
RV ESP	**20-30mmHg**
VCI diameter	**< 20mm**
